# Postprandial Endotoxin Transporters LBP and sCD14 Differ in Obese vs. Overweight and Normal Weight Men during Fat-Rich Meal Digestion

**DOI:** 10.3390/nu12061820

**Published:** 2020-06-18

**Authors:** Fabienne Laugerette, Cécile Vors, Maud Alligier, Gaëlle Pineau, Jocelyne Drai, Carole Knibbe, Béatrice Morio, Stéphanie Lambert-Porcheron, Martine Laville, Hubert Vidal, Marie-Caroline Michalski

**Affiliations:** 1 Univ Lyon, CarMeN Laboratory, INRAE, UMR1397, INSERM, UMR1060, Université Claude Bernard Lyon 1, 69310 Pierre Bénite, France; cecile.vors@univ-lyon1.fr (C.V.); maud.alligier@chu-lyon.fr (M.A.); gaelle.pineau@gmail.com (G.P.); jocelyne.drai@gmail.com (J.D.); carole.knibbe@insa-lyon.fr (C.K.); beatrice.morio@clermont.inra.fr (B.M.); martine.laville@univ-lyon1.fr (M.L.); hubert.vidal@univ-lyon1.fr (H.V.); marie-caroline.michalski@insa-lyon.fr (M.-C.M.); 2 Centre de Recherche en Nutrition Humaine Rhône-Alpes, Univ-Lyon, CarMeN Laboratory, Université Claude Bernard Lyon1, Hospices Civils de Lyon, CENS, FCRIN/FORCE Network, 69310 Pierre-Bénite, France; stephanie.lambert-porcheron@chu-lyon.fr; 3 Laboratoire de Biochimie, Centre Hospitalier Lyon Sud, 69600 Oullins, France; 4 Hospices Civils de Lyon, 69000 Lyon, France

**Keywords:** LBP, sCD14, postprandial kinetics, high-fat diet

## Abstract

Circulating levels of lipopolysaccharide-binding protein (LBP) and soluble cluster of differentiation 14 (sCD14) are recognized as clinical markers of endotoxemia. In obese men, postprandial endotoxemia is modulated by the amount of fat ingested, being higher compared to normal-weight (NW) subjects. Relative variations of LBP/sCD14 ratio in response to overfeeding are also considered important in the inflammation set-up, as measured through IL-6 concentration. We tested the hypothesis that postprandial LBP and sCD14 circulating concentrations differed in obese vs. overweight and NW men after a fat-rich meal. We thus analyzed the postprandial kinetics of LBP and sCD14 in the context of two clinical trials involving postprandial tests in normal-, over-weight and obese men. In the first clinical trial eight NW and 8 obese men ingested breakfasts containing 10 vs. 40 g of fat. In the second clinical trial, 18 healthy men were overfed during 8 weeks. sCD14, LBP and Il-6 were measured in all subjects during 5 h after test meal. Obese men presented a higher fasting and postprandial LBP concentration in plasma than NW men regardless of fat load, while postprandial sCD14 was similar in both groups. Irrespective of the overfeeding treatment, we observed postprandial increase of sCD14 and decrease of LBP before and after OF. In obese individuals receiving a 10 g fat load, whereas IL-6 increased 5h after meal, LBP and sCD14 did not increase. No direct association between the postprandial kinetics of endotoxemia markers sCD14 and LBP and of inflammation in obese men was observed in this study.

## 1. Introduction

Metabolic diseases, including obesity and type 2 diabetes, are associated with a chronic inflammatory state and increased plasma levels of lipopolysaccharides (LPS), also called endotoxins [1]. Endotoxins are components of the outer membrane of Gram-negative bacteria, which are dominant in the healthy human gut microbiome [1]. During lipid digestion, some endotoxins are translocated in the bloodstream, and thereby contribute to the onset and maintenance of low-grade inflammation during the postprandial phase following a fat-rich meal [2,3,4,5]. Indeed, Clemente-Postigo et al. (2012) reported an increase in the plasma level of endotoxins after a fat overload in morbidly obese humans [6]. This includes the binding of LPS to LPS-binding protein (LBP) and its transfer to the receptor CD14, present in both soluble (sCD14) and membrane-bound (mCD14) forms [7]. The activation of the LPS-LBP-CD14 complex leads to the secretion of pro-inflammatory markers and contributes to the inflammatory state [8]. LBP and sCD14 are now recognized as clinical markers of endotoxin exposure [9] and have been associated with obesity and metabolic disorders [10]. LBP is synthesized and released into the bloodstream in the presence of LPS, and is considered as a surrogate biomarker for the activation of LPS-induced innate immune response regarding its relative long half-life (24–48 h) [11]. Moreno-Navarrete et al. have also shown that the increase of plasma LBP in vivo may contribute to a vicious cycle that prevents white adipose tissue (WAT) expansion, and exacerbates the inflammatory response in WAT [12]. It was further shown that serum LBP and sCD14 are markers of Crohn’s disease [13]. The relative variations of LBP/sCD14 ratio are also considered important in the inflammation set-up [14]. Furthermore, higher plasma LBP concentrations have been observed in obesity [7,13,15] and plasma LBP concentration is associated to abdominal obesity [16]. We have recently shown that increase of plasma IL-6 is linked to a rise of LBP/sCD14 ratio in both humans [14] and mice [17]. More recently, Sakura et al. have suggested that plasma LBP concentration is associated with arterial stiffness, independently of traditional cardiovascular risk factors and especially in men with type 2 diabetes [18,19]. In children and adults, obesity and obstructive sleep apnea are also associated with increased LBP concentrations, and the presence of both conditions may enhance LBP concentration [20,21]. Concerning sCD14, it has been reported in mice that sCD14 presents protective effects in inflammatory bowel disease [22]. Therefore, the literature shows that LBP and sCD14 are important factors implicated in low-grade inflammation during metabolic diseases. We have previously demonstrated in obese men that postprandial endotoxemia is modified by ingested fat amount with a higher postprandial endotoxemia compared with normal-weight subjects after a higher fat load (40 g vs. 10 g) [23]. More recently, a study of healthy premenopausal women has suggested that the consumption of a pre-meal yogurt improves the postprandial metabolism and decreases metabolic endotoxemia, LBP and sCD14 [24]. However, the postprandial variations of LBP and sCD14 after a mixed meal with different amount of lipids remain poorly described in humans of different weight status. We thus analyzed the postprandial kinetics of both markers of endotoxin exposure in the context of two clinical trials involving postprandial tests in normal weight, overweight and obese men. We hypothesized that postprandial LBP and sCD14 after a fat-rich meal differed in obese vs. overweight and normal weight men, hence, contributing to different IL-6 responses after a fat load or OF.

## 2. Materials and Methods

### 2.1. Clinical Trials and Subjects

The present work relies on two previous clinical trials performed in the Human Nutrition Research Centers (CRNH) of Rhône-Alpes and Auvergne. The first clinical trial, called the Lipinflox study, was approved by the Ethics Committee of Lyon-Sud-Est-II and AFSSAPS and was registered at ClinicalTrials.gov (NCT01249378). The second one, called the Overfeeding Study, was approved by the Ethics Committee of Lyon (RG/FL-2005-067) and registered at clinicaltrials.gov (NCT00905892). The eligibility criteria of included subjects and postprandial metabolic explorations for both trials have been described elsewhere [23,25,26]. Briefly, in the crossover Lipinflox Study, 8 normal weight (20 < BMI < 25 kg/m^2^) and 8 obese (30 < BMI < 35 kg/m^2^ and waist circumference > 94 cm) men were submitted to a fat load of 10 g or 40 g at breakfast. The test breakfast contained 10 or 40 g of anhydrous milk fat with bread and a glass of skim milk (282 kcal and 551 kcal, respectively). Blood samples were collected from an antecubital arm vein through a catheter at baseline and at regular intervals during 5 h, following ingestion of fat load. Plasma was separated by centrifugation (1500 g, 10 min, 4 °C) and stored at −80 °C until further analysis. In the overfeeding clinical trial, 18 healthy young men (lean to overweight, BMI 25.8 ± 0.8 kg/m^2^, age 30.6 ± 2.1 years) were submitted to an overfeeding during 56 days. During this period, the subjects added to their daily diet +760 kcal/day as previously described [25]. Fasting plasma sampling was performed at Day 0 and Day 56. In this article, we focused on a subcohort of eight subjects that consumed a test meal (882 kcal) and performed a postprandial test including endotoxemia analyses, as described previously [25]. The mixed meal contained 33 g of fat (291 kcal) and was composed of 200 mL of Fortimel (enteral emulsion), 23 g of margarine, 9.4 g of butter, 1 g of olive oil, 85 g of bread, 20 g of jam and 200 g of banana. The body composition (fat and lean mass) was performed in 18 subjects. All subjects gave written consent after being informed of the nature, purpose, and possible risks of the clinical trial. Both clinical trials were approved by the ethics committee of Lyon Sud-Est, according to the French“Huriet-Serusclat” law and the Second Declaration of Helsinki.

### 2.2. Plasma sCD14 and LBP

To measure the plasma concentrations of circulating LBP and sCD14, plasma samples were assayed using sandwich ELISA kits (CliniSciences and R&D Systems; Nanterre, France), following the manufacturer’s instructions.

### 2.3. Plasma Analyses

Endotoxins were determined using the limule amoebocyte lysate assay in kinetic chromogenic conditions (Biogenic; Pérols, France) [15]. Serum high-sensitive C-reactive protein was assessed by immunonephelometry on an image analyzer (Beckman-Coulter; Villepinte, France). For the overfeeding trial, IL-6 was measured in serum using ELISA kits (Quantikine; Abingdon, UK). For Lipinflox, plasma hsIL-6 levels were measured using a sandwich Ultrasensitive ELISA kit (Invitrogen; Illkirch, France). The liver enzymes ASAT and ALAT were routinely measured during the screening visit (Pentra C400; Horiba, Kyoto, Japan) in order to exclude patients if the values were not biologically normal. In fact, as patients were submitted to a fat load, or to an overfeeding, it was important that they had no liver disease.

### 2.4. Anthropometry and Body Composition

As previously described by Alligier et al., body composition was determined before and after overfeeding by dual-energy X-ray absorptiometry (Hologic, Inc.; Bedford, MA, USA), and abdominal adipose tissue distribution by magnetic resonance imaging (Magnetom Symphonie 1.5 Tesla; Siemens AG, Munich, Germany) [25].

### 2.5. Statistical Analysis

Data are presented as means ± SEM and were analyzed with Graph Pad Prism^®^ (version 7.0, San Diego, CA, USA) and with R (version 3.6.3, Saint Louis, USA). Clinical characteristics of the subjects of the two clinical trials (before vs. after overfeeding and lean vs. obese subjects) were performed using Student’s *t*-test with Graph Pad Prism. To evaluate possible relationships among the various outcomes, Spearman correlations were performed using Graph Pad Prism Software. For the Overfeeding Study, postprandial kinetics of plasma LBP, sCD14, LBP/sCD14 ratio and IL-6 were each analyzed by two-way ANOVA for repeated measurements in both factors (time and overfeeding), followed by Bonferroni’s post hoc test, using Graph Pad Prism. For the Lipinflox study, postprandial kinetics of plasma LBP, sCD14 and IL-6 were each analyzed with a linear mixed-effects model, analogous to a 3-way ANOVA for repeated measurements, where the factors with fixed effects were time, obese status, fat load and their 2- and 3-way interactions, while the factor with random effects was the subject identifier. This analysis was carried out with R, using the lme function of the nlme package. The main effects and the interactions effects were tested using marginal (Type III) sums of squares like in GraphPad Prism. For the Lipinflox study, postprandial kinetics of plasma LBP, sCD14 and IL-6 were each analyzed with a linear mixed-effects model, where the factors with fixed effects were time, obese status, fat load and their 2- and 3-way interactions, while the factor with random effects was the subject identifier. This approach is similar to a three-way repeated-measures ANOVA but is more robust to missing data (we lacked IL6 data for one of the ten lean subjects). A posthoc analysis was carried out to assess the effect of obesity on the estimated marginal means at all time points. Specifically, the significance of the difference of the two marginal means (lean versus obese, collapsing the fat load) was assessed at each time point with a *t*-test based on the standard errors of the estimated marginal means. The five raw *p*-values obtained for each response in this posthoc analysis were adjusted using the False Discovery Rate (FDR) correction, in order to correct for multiple testing. This posthoc analysis was carried out with the emmeans package. A *p*-value (or a FDR-corrected *p*-value) lower than 0.05 was considered significant.

## 3. Results

### 3.1. Characteristics of the Subjects

The characteristics of the eight subjects, lean to overweight, from the overfeeding clinical trial (OF) and from the 16 subjects (normal weight and obese) from Lipinflox clinical trial are shown Table 1. As expected weight, BMI and waist circumference were significantly higher after OF, compared to before, and higher in the obese than in normal weight (NW) subjects. Concerning body composition, lean mass and fat mass were significantly enhanced after OF. CRP, IL-6 and LPS did not vary significantly in the OF clinical trial. CRP and IL-6 were higher in obese compared to NW subjects.

### 3.2. Postprandial Kinetics of LBP, sCD14 and IL-6 in Normal Weight and Obese Subjects (Lipinflox Study)

We evaluated the postprandial variations of plasma LBP, sCD14 and IL-6 in NW and obese subjects during 5 h after mixed meals differing only by fat amount: 10 vs. 40 g of milkfat spread on bread.

**Figure 1:**
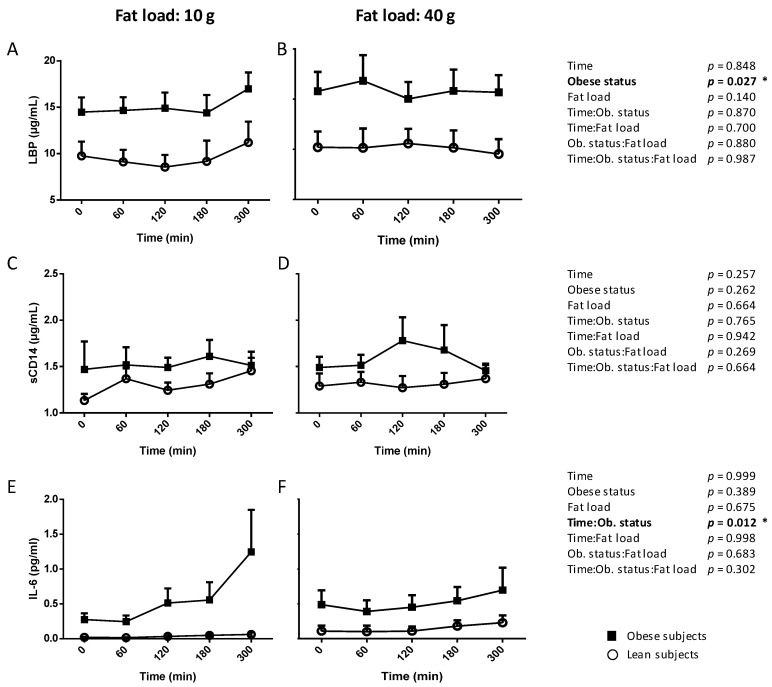
Postprandial kinetics in plasma of lipopolysaccharides (LPS)-binding protein (LBP) (**A**,**B**); sCD14 (**C**,**D**); and Il-6 (**E**,**F**) in normal-weight and obese subjects after 10 g vs. 40 g fat load. Data are means ± SEM. * *p* < 0.05.

Figure 1A,B shows that, regardless of lipid amount in the meal, the concentration of LBP in plasma was higher in obese subjects than in lean subjects (obese status, *p* = 0.027), with no impact of postprandial time. No significant difference was observed between lean and obese subjects along the postprandial kinetics of sCD14, regardless of fat load in the meal (Figure 1C,D).

Regarding IL-6, we observed an interaction effect between time and obese status (*p* = 0.012) during the postprandial kinetics (Figure 1E,F). The post-hoc analysis revealed that the significant two-way interaction between obesity and time occurred mostly at *t* = 300 min. Indeed, IL-6 plasma concentration at 300 min was on average 5.6 times higher in obese vs. lean men (*p* = 0.02) (Figure 1E).

There was no impact of BMI and postprandial time on the LBP/sCD14 ratio in all subjects for each fat amount (Appendix A).

### 3.3. Postprandial Kinetics of LBP, sCD14 and IL-6 Before and After Overfeeding

**Figure 2:**
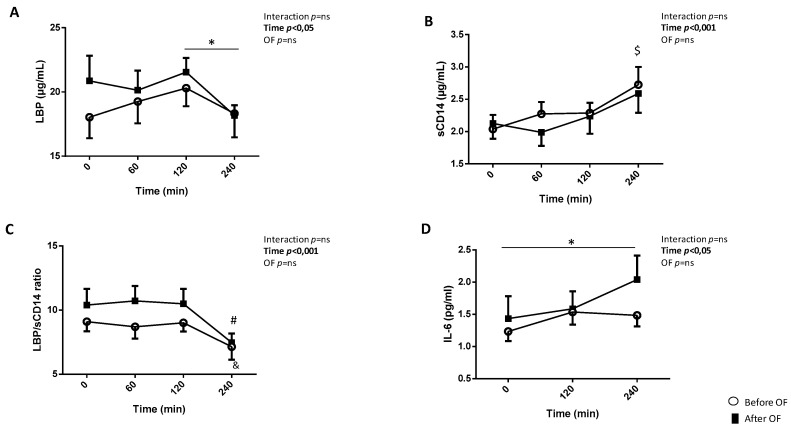
Postprandial kinetics of LBP (**A**), sCD14 (**B**), LBP/sCD14 ratio (**C**) and IL-6 (**D**) before and after overfeeding. Data are means ± SEM. The effects of time (postprandial kinetics) and overfeeding (OF) were determined by ANOVA for repeated measurements followed by post hoc test (Bonferroni). * *p* < 0.05 (after OF), & *p* < 0.05 vs. T0 and T120 (before OF); # *p* < 0.001 vs. other time (after OF); $ *p* < 0.05; 0.01; 0.001 vs. other time before and after OF.

Previously, we have shown in 18 subjects of the OF clinical trial that the LBP/sCD14 ratio was enhanced in the fasting state after OF [14]. Here, we completed our exploration by analyzing the postprandial evolution of LBP, sCD14 and LBP/sCD14 in the subgroup of eight subjects who performed the postprandial explorations. Altogether, there was no significant effect of OF on the postprandial kinetics of LBP and sCD14 (Figure 2A,B). Concerning LBP before OF, we observed a significant time effect with an increase of LBP concentration in plasma 120 min after the mixed meal (Figure 2A, *p* < 0.05). The concentration of LBP was decreased at 240 min vs. 120 min (Figure 2A, *p* < 0.05). After OF, no significant increase was observed at 120 min after the mixed meal but a significant decrease was observed at 240 min compared to 120 min (*p* < 0.05). Notably, even if after OF LBP concentration was higher at fasting compared to before OF (*p* = 0.07), at 240 min LBP concentrations were similar before and after OF (18.3 ± 1.9 µg/mL and 18.2 ± 0.8 µg/mL, respectively). A significant increase of sCD14 at 240 min compared to 0 and 60 min (*p* < 0.01 and 0.01 respectively) was observed both before and after OF (Figure 2B). The postprandial kinetics of the LBP/sCD14 ratio was not impacted by OF (Figure 2C). Both before and after OF, the LBP/sCD14 ratio decreased at 240 min (*p* < 0.001 vs. other postprandial times). We have measured IL-6 plasma concentration at 0, 120 and 240 min before and after OF (Figure 2D). There was no significant OF effect but of note, after OF plasma IL-6 concentration increased at 240 min compared to 0 min (*p* < 0.05). No interaction (between time and OF) was observed for LBP, sCD14, LBP/sCD14 and IL-6 (Figure 2A–D).

### 3.4. Associations between Fasting and Postprandial LBP and Selected Parameters of Dietary Trials and Intervention

We examined the association of plasma LBP concentrations with some parameters related to obesity and metabolic disorders in subjects from both the overfeeding and Lipinflox studies. Subjects of the Lipinflox study who have consumed a test meal with 40 g of lipids presented no significant correlation between circulating LBP and the selected parameters, age, weight, BMI, waist circumference (WC), aspartame amino transferase (AST), alanine amino transferase (ALT), sCD14 (data not shown).

The correlations between LBP and selected parameters of the Lipinflox postprandial clinical trial after the test meal containing 10 g of fat are reported Table 2. At all times of the postprandial kinetics, the LBP concentration was significantly and positively associated with waist circumference (WC), but not with weight and BMI in all subjects from Lipinflox clinical trial (lean and obese, Table 2). After stratification according to BMI group, this positive correlation was maintained for lean subjects, but not for obese subjects (Table 2). LBP concentration was also significantly and positively associated with ALT but not with AST. These correlations were also maintained for lean subjects at 0, 60 and 120 min, a trend was observed at 180 and 300 min (Table 2). Conversely, no association of LBP with WC or with ALT was observed for obese subjects (Table 2).

Concerning the overfeeding clinical trial, we also used some mass parameters, such as abdominal visceral fat and subcutaneous WAT depots, measured by magnetic resonance imaging [27]. As shown in Table 3, LBP concentration in plasma was significantly and positively associated with trunk lean mass, lean mass and waist circumference before and after OF. No correlation with subject age was observed (data not shown). Notably, the LBP/sCD14 ratio was significantly and positively associated with lean mass, fat trunk and BMI, before and after overfeeding (Table 3).

## 4. Discussion

The novel feature of the present study is the postprandial explorations of LBP and sCD14 concentrations in plasma in two different clinical trials in obese vs. overweight and normal-weight men. In the first clinical trial (Lipinflox), we have shown that obese men presented a higher LBP concentration in plasma than lean men, regardless of fat load (10 g vs. 40 g), with no time effect during the postprandial period. However, no significant differences in sCD14 concentrations were observed between lean and obese men. In the second clinical trial (overfeeding), we demonstrated, in lean to overweight men, a time effect on LBP, sCD14, IL-6 concentrations and LBP/sCD14 ratio during the postprandial period. Only a few human studies have focused on the effect of an OF on endotoxemia [24] and, to our knowledge, no study has been performed to date in order to evaluate the impact of dietary fat intake on the postprandial kinetics of LBP and sCD14 in NW, overweight and obese men.

It is now well established that the intestinal barrier function is modified in obesity, leading to low-grade inflammation and endotoxemia [9]. Endotoxins have been described as contributors to the inflammation observed in hypercaloric diets [5,28,29]. LBP and sCD14, two important actors in the endotoxin metabolic pathway, are now considered indirect markers of gut permeability in metabolic diseases, but also in other diseases, such as HIV [9,22]. Indeed, both reductions of these markers and gut permeability were shown after 12 weeks of fish oil supplementation, compared to placebo in HIV+ patients [30]. We show in the Lipinflox study that LBP concentration was higher in obese men at all times of the postprandial kinetics, independently of fat load (10 vs. 40 g of fat).

The relationship between inflammation and “metabolically healthy” and “non-metabolic healthy” status remains relatively unknown, even though some studies have shown relationships between inflammatory markers and obesity or metabolic syndrome. Aguilar-Salinas CA et al. demonstrated that obese individuals depict similar adiponectin levels to normal-weight subjects and this may be associated with the “metabolically healthy” obese phenotype [31]. In turn, patients with severe obesity were reported to have higher LBP concentration, which can be reduced after bariatric surgery [32]. In a recent study, it was suggested that serum LBP is related to abdominal obesity more than to metabolic health [16]. Serum LBP is also known to be associated with the carotid intima media thickness [19], suggesting that the pro-inflammatory action of LBP might be a contributor to the progression of cardiovascular events [11,33]. In hemodialysis patients, for whom gut barrier and microbiota are impaired, a positive association between LBP and chronic inflammation and metabolic syndrome was reported [34]. The present study shows a strong relationship between plasma LBP and waist circumference. It is interesting to note that plasma LBP was correlated significantly with WC only in lean subjects. Another point is the correlation of LBP with ALT only in lean subjects. Patients with non-alcoholic fatty liver disease are known to present higher LBP concentrations when they develop steatohepatitis [15]. An association between LBP and ALT was noted in hepatitis C virus, and these authors suggested that, as with ALT, LBP might serve as another hepatic inflammatory biomarker [35]. Another study has shown that endotoxemia reflects the hepatic functional reserve capacity of end-stage liver disease [36]. Interestingly, in obese subjects of the present study, no correlation of circulating LBP with WC or ALT was observed. Naghizadek et al., 2018 have shown that WC is important for the association among TLR4, serum LBP and IFNβ and metabolic state only for the highest WC range [16]. This finding is not in agreement with the present study, and could be explained by the fact that obese subjects were not morbid.

In the Lipinflox study, the fat load (10 vs. 40 g) did not modify the kinetics of the LBP concentration in the plasma of lean and obese subjects. However, the LBP concentration in lean subjects was lower than that in obese subjects for both fat loads. Moreover, the dietary intervention in the OF study did not affect postprandial LBP concentrations. Umoh F. et al. also suggested that obesity might not result in enhanced exposure to intestinal bacteria, as the effect of BMI was no longer significant in multiple linear regression models [37]. It is well known that obesity is associated with modifications in the secretion of cytokines from adipose tissue and liver. However, only few studies have investigated the impact of overfeeding on the production of cytokines/adipokines. In the present OF study, the concentrations of LBP, sCD14 and Il-6 were not significantly affected by a two-month overfeeding (+760 kcal/day by adding 70 g of lipids to the usual daily diet). More precisely, the fat OF consisted in daily addition of 20 g of butter, 100 g of cheese (Emmental) and 40 g of almonds. Another study of acute overfeeding (+1250 kcal/day with a nutrient composition of 45% fat, 15% protein and 40% carbohydrate) has shown that 3 days are sufficient to increase body weight and HOMA-IR, without affecting MCP-1 and CRP plasma concentrations [38]. Tam C.S. et al. also demonstrated that moderate weight gain after 28 days of overfeeding (+1250 kcal via high-fat snacks composed of 45% fat, 15% protein, and 40% carbohydrate) in healthy humans resulted in a significant weight gain and increased circulating levels of CRP and MCP-1, without changes in subcutaneous WAT mass [39]. Those results demonstrate that different compositions of overfeeding diets may differentially impact cytokines/adipokine concentrations in plasma and tissues.

The present study has some limitations. Firstly, the sample size was small, owing the cumbersome aspects of postprandial explorations, and only one plasma inflammatory marker was measured. However, the postprandial results were obtained from two different trials, and the timing of plasma collection was not the same. Another limitation of this study is the measure of IL-6 in plasma taken serially from a catheter in an arm vein. Indeed some authors have shown that the presence of the catheter irritate the vein and cause an increase in IL-6 levels in plasma samples taken over several hours compared to fresh samples taken from the opposite arm [40,41]. Finally, ensuring adherence to dietary instructions given during the OF clinical trial is difficult in a feeding trial.

To conclude, we have shown in obese men that a fat load of 10 g of lipids can drive an increase of IL-6 concentration in plasma at 300 min post-meal, maybe due to a lesser clearance of LPS by lipoproteins. Indeed Vors et al. has shown that obese subject chylomicrons were more enriched with LPS compared to NW, which could contribute to LPS clearance and to a lesser IL-6 concentration in plasma [23]. This result should be confirmed in further studies with the measure of other inflammatory markers. The LBP concentration in plasma was higher in obese subjects than in NW subjects both at fasting and along the postprandial period (Lipinflox clinical trial), and was significantly and positively associated with trunk lean mass, lean mass and waist circumference before and after OF. Further studies are now necessary to better understand the relative role of LBP and sCD14 during the postprandial phase after diets containing different amounts and types of fats in different food matrixes.

## Figures and Tables

**Table 1:** Clinical characteristics of study subjects. Data are means ± SEM. Groups are compared using unpaired Student *t*-test, * *p* < 0.05, ** *p* < 0.01; *n* = 8 OF clinical trial, *n* = 8 normal weight, *n* = 8 obese.

	Overfeeding Clinical Trial			Lipinflox Clinical Trial		
	Before OF	After OF	*p* Value	Normal Weight	Obese	*p* Value
Age (year)	26 ± 2			29 ± 1	31 ± 2	0.426
Body weight (kg)	78.9 ± 4.9	81.7 ± 4.9	0.005 **	72.5 ± 2.1	101.1 ± 2.1	0.001 **
BMI (kg/m^2^)	24.9 ± 1.5	25.7 ± 1.4	0.004 **	22.4 ± 0.5	31.8 ± 0.3	0.001 **
Waist circumference (cm)	84.9 ± 3.3	86.9 ± 3.3	0.038 *	83.6 ± 1.7	105 ± 0.8	0.001 **
hsCRP (µg/mL)	0.6 ± 0.1	1.1 ± 0.5	0.38	1.96 ± 0.01	2.98 ± 0.47	0.036 *
IL-6 (pg/mL)	1.23 ± 0.15	1.43 ± 0.35	0.53	0.18 ± 0.04	0.45 ± 0.16	0.006 *
LPS (EU/mL)	0.11 ± 0.03	0.16 ± 0.10	0.59	0.19 ± 0.05	0.18 ± 0.04	0.729
Lean mass (kg)	57,3 ± 2.5	58.2 ± 2.60	0.032 *	-	-	
Fat mass (kg)	15.8 ± 2.9	17.1 ±3.1	0.048 *	-	-	
Visceral fat (kg)	66.0 ± 18.1	73.5 ± 16.5	0.469	-	-	

**Table 2:** Correlation analyses (Spearman) with postprandial plasma LBP concentrations as dependent variable after a 10 g fat load (Lipinflox clinical trial in men). * *p* < 0.05; ** *p* < 0.01.

	All (*n* = 16)									
Postprandial Time	0 min		60 min		120 min		180 min		300 min	
	r	*p*	r	*p*	r	*p*	r	*p*	r	*p*
**Age (years)**	0.66	ns	−0.06	ns	0.065	ns	−0.10	0.07 ns	0.23	ns
**Weight (kg)**	0.26	ns	0.42	ns	0.43	0.058	0.37	ns	0.36	ns
**BMI (kg/m^2^)**	0.24	ns	0.35	ns	0.44	0.052	0.37	ns	0.36	ns
**Waist circumference (cm)**	**0.58**	0.007 **	**0.58**	0.006 **	**0.63**	0.003 **	**0.41**	0.07	**0.51**	0.02 *
**AST (U/L)**	0.20	ns	0.1	ns	0.24	ns	0.27	ns	0.14	ns
**ALT (U/L)**	**0.49**	0.02 *	**0.57**	0.008 **	**0.63**	0.003 **	**0.53**	0.016 *	**0.43**	0.05 *
**sCD14 T0 (µg/mL)**	0.41	0.06	**0.45**	0.04 *	0.28	ns	−0.15	ns	0.058	ns
	**Lean (*n* = 8)**									
	T0 min		T60 min		T120 min		T180 min		T300 min	
	r	*p*	r	*p*	r	*p*	r	*p*	r	*p*
**Age (years)**	**0.7**	0.02 *	0.43	ns	0.43	ns	0.35	ns	0.47	ns
**Weight (kg)**	−0.31	ns	−0.16	ns	−0.02	ns	0.09	ns	0.07	ns
**BMI (kg/m^2^)**	−0.23	ns	−0.18	ns	−0.08	ns	0.02	ns	0.05	ns
**Waist circumference (cm)**	**0.66**	0.04 *	**0.60**	0.06	**0.71**	0.02 *	**0.72**	0.02 *	**0.58**	0.08
**AST (U/L)**	0.55	ns	0.18	ns	0.22	ns	0.27	ns	0.23	ns
**ALT (U/L)**	**0.69**	0.03 *	**0.62**	0.05 *	**0.68**	0.03 *	0.51	ns	0.38	ns
**sCD14 T0 (µg/mL)**	0.05	ns	0.05	ns	−0.01	ns	0.17	ns	−0.02	ns
	**Obese (*n* = 8)**									
	T0 min		T60 min		T120 min		T180 min		T300 min	
	r	*p*	r	*p*	r	*p*	r	*p*	r	*p*
**Age (years)**	−0.48	ns	−0.56	ns	−0.17	ns	−0.09	ns	−0.04	ns
**Weight (kg)**	−0.13	ns	0.18	ns	−0.27	ns	0.25	ns	0.06	ns
**BMI (kg/m^2^)**	−0.31	ns	−0.46	ns	−0.13	ns	0.07	ns	0.17	ns
**Waist circumference (cm)**	0.25	ns	0.05	ns	0.13	ns	−0.13	ns	0.35	ns
**AST (U/L)**	−0.33	ns	−0.27	ns	−0.07	ns	0.23	ns	−0.13	ns
**ALT (U/L)**	−0.11	ns	0.04	ns	0.18	ns	0.53	ns	0.16	ns
**sCD14 T0 (µg/mL)**	0.22	ns	0.4	ns	0.10	ns	−0.13	ns	−0.29	ns

**Table 3:** Correlation analyses (Spearman) of fasting LBP and LBP/sCD14 with mass parameters in the overfeeding clinical trial (*n* = 18 men). * *p* < 0.05; ** *p* < 0.01.

Before OF			
Variable 1	Variable 2	r	*p*
LBP (µg/mL)	Trunk lean mas (kg)	0.65	0.002 **
	Lean mass (kg)	0.57	0.009 **
	Waist circumference (cm)	0.53	0.02 *
	BMI (kg/m^2^)	0.47	0.04 *
	Fat trunk (kg)	0.41	ns
	Visceral fat (kg)	0.52	0.03 *
LBP/sCD14	Lean mass (kg)	0.62	0.004 **
	Fat trunk (kg)	0.48	0.04 *
	BMI (kg/m^2^)	0.62	0.004 **
**After OF**			
Variable 1	Variable 2	r	*p*
LBP (µg/mL)	Trunk lean mas (kg)	0.62	0.003 **
	Lean mass (kg)	0.56	0.01 *
	Waist circumference (cm)	0.51	0.02 *
	BMI (kg/m^2^)	0.32	ns
	Fat trunk (kg)	0.47	0.04 *
	Visceral fat (kg)	0.07	ns
LBP/sCD14	Lean mass (kg)	0.49	0.03 *
	Fat trunk (kg)	0.49	0.03 *
	BMI (kg/m^2^)	0.49	0.03 *

## Abbreviations

ALT, alanine amino transferase; AST, aspartame amino transferase; LBP, lipopolysaccharides-binding protein; LPS, lipopolysaccharides; NW, normal weight; OF, overfeeding; sCD14, soluble cluster of differentiation; WAT, white adipose tissue; WC, waist circumference.
